# Repression of RNA Polymerase II Transcription by B2 RNA Depends on a Specific Pattern of Structural Regions in the RNA

**DOI:** 10.3390/ncrna1010004

**Published:** 2015-01-28

**Authors:** Steven L. Ponicsan, Jennifer F. Kugel, James A. Goodrich

**Affiliations:** University of Colorado, Department of Chemistry and Biochemistry, 596 UCB, Boulder, CO 80309, USA; E-Mail: steven.ponicsan@colorado.edu

**Keywords:** non-coding RNA, transcription, RNA polymerase II, B2 RNA, repressor, SINE

## Abstract

B2 RNA is a mouse non-coding RNA that binds directly to RNA polymerase II (Pol II) and represses transcription by disrupting critical interactions between the polymerase and promoter DNA. How the structural regions within B2 RNA work together to mediate transcriptional repression is not well understood. To address this question, we systematically deleted structural regions from B2 RNA and determined the effects on transcriptional repression using a highly purified Pol II* in vitro* transcription system. Deletions that compromised the ability of B2 RNA to function as a transcriptional repressor were also tested for their ability to bind directly to Pol II, which enabled us to distinguish regions uniquely important for repression from those important for binding. We found that transcriptional repression requires a pattern of RNA structural motifs consisting of an extended single-stranded region bordered by two stem‑loops. Hence, there is modularity in the function of the stem-loops in B2 RNA—when one stem‑loop is deleted, another can take its place to enable transcriptional repression.

## 1. Introduction

Non-coding RNAs (ncRNAs) are now understood to be active participants in regulating the process of transcription [1,2]. One such ncRNA transcriptional regulator is mouse B2 RNA. B2 RNA is a family of ~180 nucleotide RNAs that are transcribed by RNA polymerase III (Pol III) from SINEs that are abundant in the mouse genome [3]. Although SINEs have typically been considered “junk DNA” we found that B2 RNA acts as a repressor of mRNA transcription by Pol II after heat shock, thereby revealing a mechanism by which mRNA genes can be repressed during a cellular stress as well as identifying a function for SINE RNAs [4].

Biochemical studies have provided insight into the mechanism by which B2 RNA functions as a transcriptional repressor. B2 RNA binds directly to core Pol II with high affinity and kinetic stability [5]. Mass spectrometry and cryo EM studies revealed that B2 RNA occupies the DNA binding cleft of Pol II [6,7]. Despite this, when B2 RNA is bound, Pol II is able to interact with general transcription factors and assemble into complexes on promoter DNA [5]. In these complexes, the majority of polymerase/promoter contacts are disrupted [8]. It is likely that a network of protein-protein and protein-DNA interactions hold complexes containing Pol II and B2 RNA on the promoter even when Pol II is unable to directly bind the promoter DNA.

**Figure 1:**
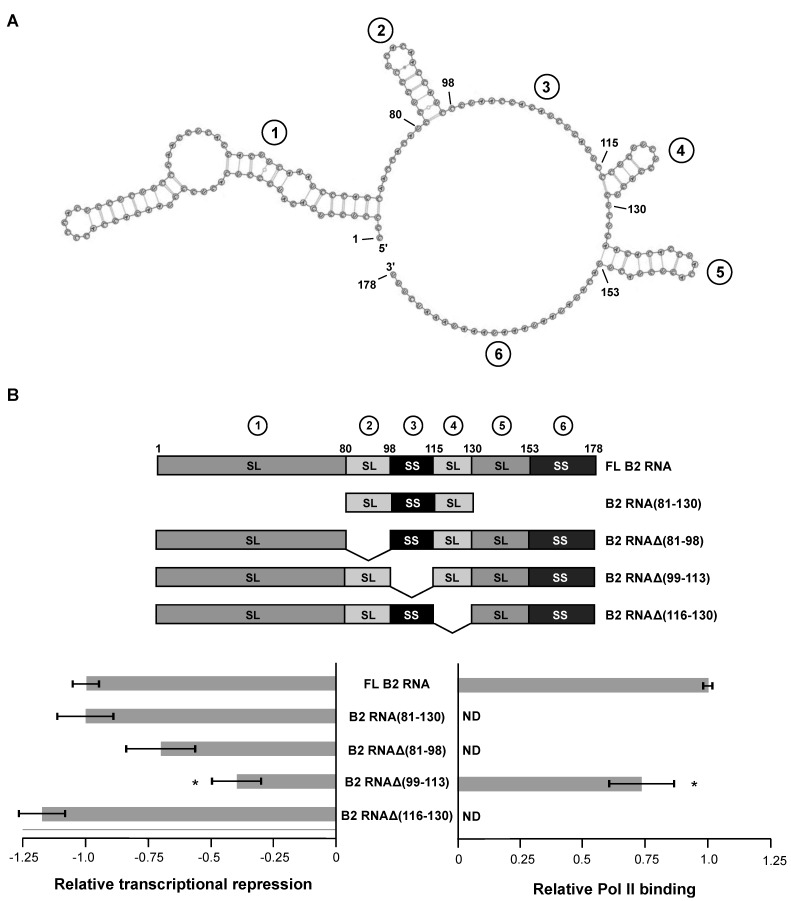
Internal deletion of structural regions from B2 RNA shows that the single-stranded region 3 is required for transcriptional repression. (**A**) B2 RNA has six distinct structural regions. The experimentally determined secondary structural model of B2 RNA is shown with the six structural regions numbered. The image was generated using VARNA software [10]; (**B**) Single-stranded region 3 is required for transcriptional repression, whereas the stem-loops in regions 2 and 4 are not. Relative transcriptional repression for each deletion compared to full length B2 RNA is shown, with zero representing no repression. The data are the average of minimally 3 replicates and the error bars are the standard error of the mean. The asterisk indicates a statistically significant difference (*p* value < 0.05) between a deletion construct and full-length B2 RNA. The deletion constructs are illustrated in the schematic beside the plot.

We previously determined a model for the secondary structure of B2 RNA using RNase protection assays and in-line probing, which identified six distinct structural regions (Figure 1A, regions 1–6) [9]. The structure can be summarized as containing 4 stem-loops (SL, regions 1, 2, 4, and 5) and two extended, single-stranded regions (SS, regions 3 and 6). Our previous studies found the minimal region of B2 RNA that was functional for high affinity binding to Pol II and transcriptional repression spanned nucleotides 81–130 (regions 2–4) [9]. In the context of this minimal construct, we found that nucleotides 81–98 (region 2) were required for transcriptional repression, but not Pol II binding. Moreover, nucleotides 99–115 (region 3) must remain single stranded for the minimal construct to function as a transcriptional repressor, but this was not important for binding Pol II. Lastly, nucleotides 116–130 (region 4) were required for Pol II binding, and hence also transcriptional repression. We did not, however, determine the roles of these three regions, or other regions, in the context of the full length B2 RNA.

To better understand the mechanism of transcriptional repression by B2 RNA, we examined the contribution of all six structural region(s) to the repression of Pol II transcription. To do so, we took a two-pronged approach. First, we internally deleted from full length B2 RNA the 3 regions we previously found to be important in the context of a minimal functional piece of B2 RNA. Second, we serially deleted each of the distinct structural regions from the 5’ and 3’ ends of B2 RNA and evaluated the effect on transcriptional repression* in vitro*. Our results show that a single-stranded region in B2 RNA is required for transcriptional repression and this region must be flanked by stem-loops on both sides. The stem-loops on either side of the single-stranded region function in a modular manner. Lastly, our data reinforce the model that Pol II binding and transcriptional repression by B2 RNA are separable functions.

## 2. Results

### 2.1. Deleting Individual, Internal Structural Regions from Full-Length B2 RNA Reveals Only One Region That Is Important for Transcriptional Repression

We previously found that structural regions 2, 3, and 4 were each important for transcriptional repression and/or Pol II binding when perturbed in the context of the minimal, functional B2 RNA construct (B2 RNA(81–130) [9]. We first determined how each of these three domains functioned in the context of the full length B2 RNA. To do so we generated B2 RNA constructs each lacking one of the three regions, and assayed the mutant RNAs for their ability to repress transcription* in vitro*. The transcription assays contained highly purified human Pol II, the general transcription factors TBP, TFIIB, and TFIIF, and a strong core promoter contained on a negatively supercoiled plasmid. The promoter was upstream of a G-less cassette that allowed an RNA product of defined length (390 nt) to be transcribed in the presence of ATP, CTP, and UTP. This product was resolved on a gel and quantified.

Figure 1B shows the quantitation of transcription data obtained in the absence and presence of five different B2 RNA constructs: full length B2 RNA, the minimal B2 RNA(81–130) that is functional for repression, and three internal deletions, including B2 RNAΔ(81-98), B2 RNAΔ(99–113), and B2 RNAΔ(116–130). In the case of B2 RNAΔ(99–113), the entire single stranded region (99–115) was not deleted; instead, two nucleotides in the unstructured region remained to serve as a linker between the flanking stem-loops. The data were normalized such that repression by full-length B2 RNA was set to −1.0. Hence, in the plot on the left, zero represents the un-repressed state. The data shown for full-length B2 RNA is the average from all experiments and is shown in all figures hereafter; full length B2 RNA repressed transcription 7.8-fold on average. Representative raw transcription data for the constructs tested in Figure 1B are shown in Figure S1A. As we had previously observed, B2 RNA(81–130) repressed transcription as well as full-length B2 RNA. Surprisingly, of the three internal deletion mutants, only B2 RNAΔ(99–113), which lacked the extended single-stranded region 3, showed a significant reduction in transcriptional repression (indicated by the asterisk). B2 RNAΔ(81–98), which lacked the stem-loop region 2, was slightly weaker than full-length B2 RNA in its ability to repress transcription, but the difference was not statistically significant. B2 RNAΔ(116–130), which lacked the stem-loop region 4, was as potent a transcriptional repressor as full-length B2 RNA.

Prior to concluding that the 99-113 region of B2 RNA is important in mediating transcriptional repression, we asked whether the impaired ability of B2 RNAΔ(99–113) to repress transcription was due to impaired ability to bind to Pol II. The other B2 RNA deletions, which maintained full ability to repress transcription were not tested in binding assays (indicated by ND, in the plot on the right), since all previous experiments that we have done with ncRNA repressors have conclusively shown that full repression requires high affinity binding to Pol II [5,9,11]. To evaluate binding, ^32^P‑labeled full length B2 RNA and B2 RNAΔ(99–113) were incubated with Pol II, and the mixtures were run on native gels to resolve bound RNA from unbound RNA. These electrophoretic mobility shift assays were quantitated to determine the amount of RNA bound to Pol II, and the data were normalized to binding by full-length B2 RNA, which was set to a value of 1.0. Representative raw binding data for the constructs tested in Figure 1B are shown in Figure S1B. As shown in the plot on the right side of Figure 1B, there was a small but statistically significant reduction in the capacity of B2 RNAΔ(99–113) to bind to Pol II compared to full length B2 RNA. Importantly, however, the reduction was not large enough to account for the impact that deleting 99–113 had on transcriptional repression. Hence, we conclude that single-stranded region 3 is important for mediating transcriptional repression in the context of full length B2 RNA.

The observations in Figure 1B were surprising given our earlier studies, and suggest that the structure-function relationships in full length B2 RNA are more complex than in a minimal functional piece of the RNA. Specifically, deletion of the stem-loop in region 2 from full length B2 RNA did not cause a significant reduction in transcriptional repression, whereas deleting it from B2 RNA(81–130) abolished transcriptional repression [9]. Moreover, deletion of the stem-loop in region 4 from full length B2 RNA did not cause a reduction in transcriptional repression, whereas deleting it from B2 RNA(81–130) abolished transcriptional repression and Pol II binding [9]. Together these observations suggest it is possible that other regions of B2 RNA could function in mediating the repression of Pol II transcription, and moreover, these regions could function redundantly with regions 2 and 4 of B2 RNA. For example, deletion of the region 2 stem-loop effectively replaced it with the region 1 stem-loop, and deletion of the region 4 stem-loop effectively replaced it with the region 5 stem-loop. Perhaps transcriptional repression requires that stem-loop structures flank single-stranded region 3, but the nature of those structures is modular. In this model, the stem-loops in regions 1 and 2 act redundantly, and the stem-loops in regions 4 and 5 act redundantly.

### 2.2. Serially Deleting Structural Regions from the 5’ End or 3’ End of B2 RNA Reveals a Structural Pattern That is Required for Transcriptional Repression

**Figure 2:**
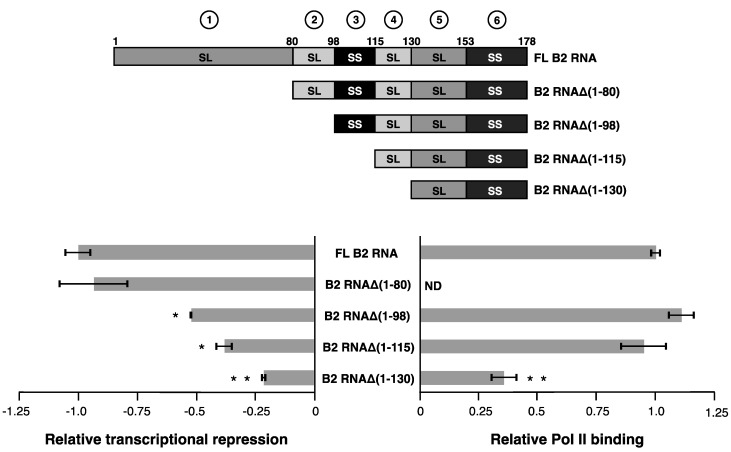
Serially deleting structural regions from the 5' end of B2 RNA shows that the stem-loop in region 2 functions in transcriptional repression. Relative transcriptional repression is shown in the plot on the left; zero represents no repression. The plot on the right shows binding to Pol II, where a value of 1.0 on the X-axis represents complete binding and ND indicates not tested in binding assays. A single asterisk indicates a statistically significant difference in transcriptional repression between the deletion construct and full-length B2 RNA (*i.e.*, a *p* value < 0.05). Two asterisks (* *) indicate that the deletion construct is significantly different from both full-length B2 RNA and its next longest neighbor. For example, B2 RNAΔ(1–130) is significantly different from both full-length B2 RNA, and B2 RNAΔ(1-115). The deletion constructs are illustrated in the schematic above the plot.

As a strategy to test the model proposed above we systematically truncated B2 RNA by serially removing structural regions from the 5’ end or the 3’ end, and then determined the effect on transcriptional repression. A schematic of the deletion mutants that removed structural regions from the 5’ end is shown at the top of Figure 2. The impact of the deletions on transcriptional repression is shown in the plot on the bottom left of Figure 2. Removing structural region 1 to generate B2 RNAΔ(1–80), minimally impacted the ability of B2 RNA to repress transcription. Further removal of the stem-loop region 2, generating B2 RNAΔ(1–98), significantly decreased the ability to repress transcription. B2 RNAΔ(1–98) remained fully functional in Pol II binding (Figure 2, right plot), allowing us to conclude that region 2 indeed contributes to transcriptional repression. Taken together with the data in Figure 1B, we conclude that the region 1 stem-loop and the region 2 stem-loop can act redundantly in mediating transcriptional repression—one, or both, of these regions needs to be present for B2 RNA to repress Pol II transcription. Moreover, when both regions were deleted, the resulting B2 RNAΔ(1–98) binds Pol II as well as full-length B2 RNA, which shows that transcriptional repression can be separated from Pol II binding, and implying that regions 1 and 2 can act as transcriptional repression domains.

We continued deletions from the 5’ end to determine if other regions were important for transcriptional repression, or Pol II binding. The two remaining B2 RNA constructs that serially deleted regions 3 then 4, B2 RNA (B2 RNAΔ(1–115) and B2 RNAΔ(1–130)), were each significantly impaired in their ability to repress transcription when compared to full-length B2 RNA, as expected. To specifically ask if regions 3 and 4 contribute to repression, we compared each deletion to its next longest neighbor in the deletion series. Only the difference in repression between B2 RNAΔ(1–130) and B2 RNAΔ(1–115), its next longest neighbor, was significant (indicated by two asterisks), suggesting region 4 was important for repression. However, as shown in the plot on the right side of Figure 2, B2 RNAΔ(1–130)) was significantly impaired for binding to Pol II. Therefore, we conclude that region 4 contributes to binding to Pol II, which is consistent with our earlier observations in the context of B2 RNA(81–130) [9].

**Figure 3:**
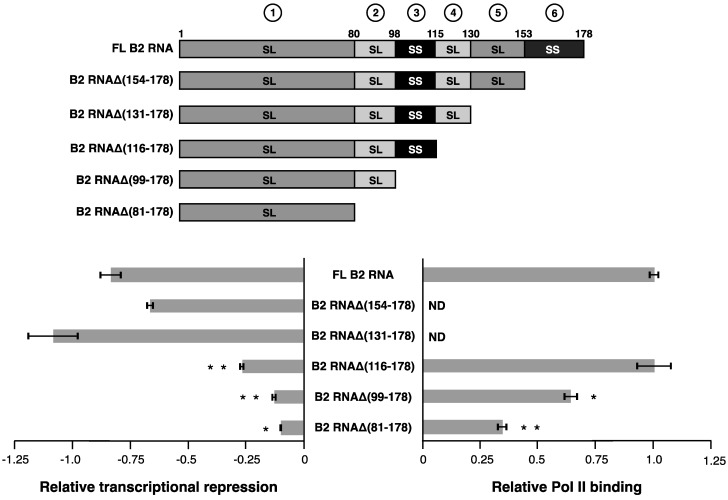
Serially deleting structural regions from the 3’ end of B2 RNA shows that two regions (3 and 4) contribute to transcriptional repression. See the legend in Figure 2 for a description of the plots and labeling.

We performed an analogous set of experiments, deleting structural regions from the 3’ end of B2 RNA (diagrammed at the top of Figure 3) and testing their ability to repress Pol II transcription. Removing the unstructured A/U rich tail in region 6 (nucleotides 154–178) and the stem-loop in region 5 (nucleotides 131–153) did not decrease transcriptional repression (bottom left plot of Figure 3). The first statistically significant decrease in the capacity to repress transcription occurred as the stem-loop encompassing nucleotides 116–130 (region 4) was additionally deleted to form B2 RNAΔ(116–178). This decrease in the ability to repress transcription was significant both compared to the full-length RNA and to its next longest neighbor, B2 RNAΔ(131–178). Moreover, B2 RNAΔ(116–178) was fully functional in Pol II binding (Figure 3, right plot). Taken together with the data in Figure 1B, we conclude that the region 4 stem-loop and the region 5 stem-loop can act redundantly in mediating transcriptional repression – one, or both, of these regions need to be present for B2 RNA to repress Pol II transcription. Importantly, neither region is required for Pol II binding, showing that transcriptional repression can be separated from Pol II binding, and implying that regions 4 and 5 can act as transcriptional repression domains.

Continuing with the 3’ end deletion series, an additional impact on repression was observed as the single-stranded region from 99 to 115 (region 3) was removed, generating B2 RNAΔ(99–178). B2 RNAΔ(99–178) showed a slight but statistically significant reduction in Pol II binding; however this reduction was not enough to explain the entire effect of the deletion on transcriptional repression. This is consistent with the earlier observations that single-stranded region 3 is important for transcriptional repression in the context of both full length B2 RNA (Figure 1B) and B2 RNA(81–130) [9]. Lastly, the additional truncation of region 2 to generate B2 RNAΔ(81–178) had little impact compared to its next longest neighbor, but this RNA remained impaired for repression compared to full-length RNA as expected. B2 RNAΔ(81–178) was reduced in its ability to bind to Pol II to a similar extent as the reduction in its ability to repress transcription.

### 2.3. The Ability to Repress Transcription Does not Correlate with RNA Length

**Figure 4:**
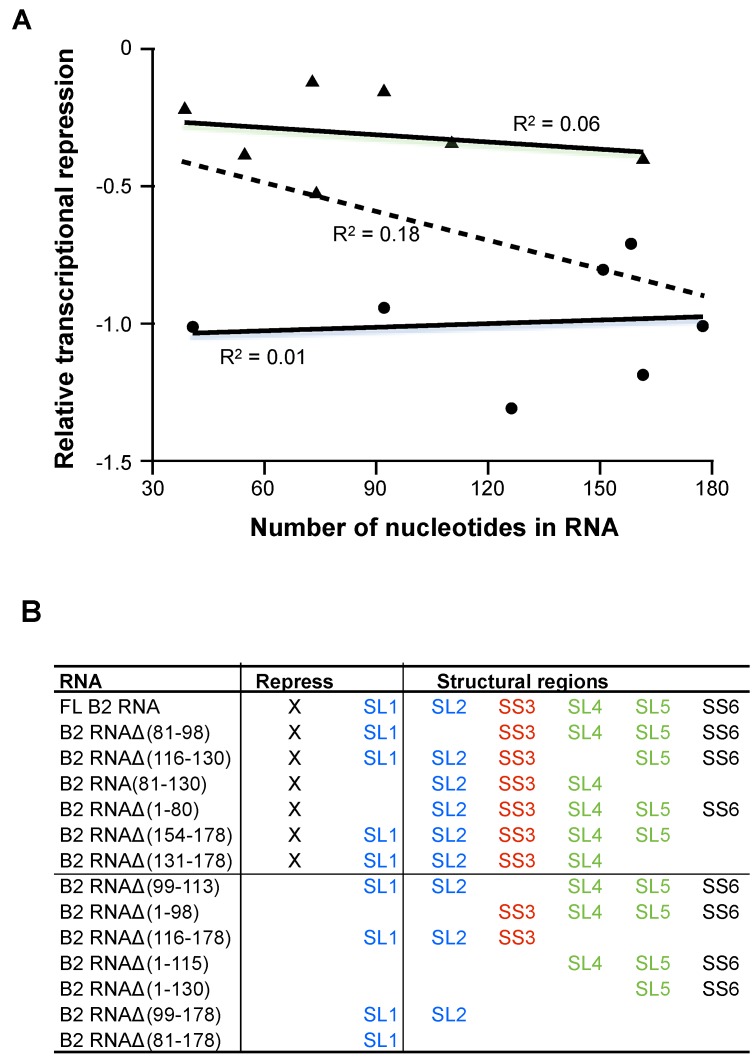
Transcriptional repression does not correlate with RNA length. A) The number of nucleotides in each B2 RNA construct (full length and each of 13 deletions) was plotted against its level of transcriptional repression (relative to full length, which was set to −1). The circles represent full-length B2 RNA and deletions that showed a level of transcriptional repression that was not significantly reduced from the level of repression observed with the full-length RNA. The triangles represent RNA deletions that were significantly impaired for transcriptional repression. Fitting all of the data using linear regression resulted in an *R*^2^ value of 0.18. Fitting just the data represented by circles and triangles yielded *R*^2^ values of 0.01 and 0.06, respectively; (**B**) Summary of the transcriptional repression activity, the Pol II binding activity, and the patterns of structural regions in B2 RNA constructs. The stem loops in blue are interchangeable for transcriptional repression, as are the stem loops in green. The red single-stranded region is required for repression, and the black single stranded region is dispensable.

In analyzing the B2 RNA deletions for transcriptional repression, we noticed a trend that the least potent transcriptional repressor RNAs were in general shorter RNAs. Hence, we questioned whether the repressive ability of an RNA would simply correlate with its size. To evaluate this for full-length B2 RNA and each of the 13 deletion RNAs shown in Figure 1, Figure 2 and Figure 3, the length of the RNA in nucleotides was plotted* versus* the potency of transcriptional repression. As shown by the dashed line in Figure 4A (fit through all 14 points), the level of transcriptional repression does not correlate with RNA size (*R*^2^ = 0.18). We further tested this by separately considering the RNAs that potently repressed transcription (plotted as circles) and the RNAs that were impaired for transcriptional repression (plotted as triangles). In both cases there was no correlation between the level of transcriptional repression and RNA size, as shown by the two solid lines and corresponding *R*^2^ values.

## 3. Discussion

We studied the extent to which distinct structural regions in B2 RNA contribute to the repression of Pol II transcription. Internal deletion of individual structural regions from B2 RNA showed that the single-stranded region spanning nucleotides 99–115 (region 3) makes the greatest contribution to transcriptional repression. We were surprised to learn that individually deleting the stem-loops on either side of this single-stranded region (*i.e.*, regions 2 and 4) did not affect transcriptional repression, because deleting these stem-loops in the context of a minimal functional piece of B2 RNA had strong effects [9]. In the context of full length B2 RNA, however, deletion of either stem-loop 2 or 4 resulted in a more distal stem-loop moving in to flank the single-stranded region, suggesting that the stem-loops surrounding the single-stranded region function in a modular manner. To further test this model, we serially deleted structural regions from the 5’ and 3’ ends of B2 RNA, and learned that in all cases single-stranded region 3 must be bordered on both sides by a stem-loop in order to repress transcription. In addition, partial B2 RNA constructs were identified that bound Pol II similarly to full-length B2 RNA, but did not fully repress transcription. This reinforces the model that Pol II binding, while required for transcriptional repression by B2 RNA, is not in itself sufficient for transcriptional repression.

To function as a repressor, B2 RNA utilizes a structural pattern that is composed of two stem-loops (SL) interrupted by an extended single stranded region (SS). As shown in Figure 4B, all of the RNA constructs that repress transcription contain the SL-SS-SL pattern, whereas none of the RNA constructs that are impaired for repression contain the SL-SS-SL pattern. Although the pattern is important, the identity of the stem-loops is not important; either of two stem-loops (region 1 or region 2) on the 5’ side of single-stranded region 3, and either of two stem-loops (region 4 or region 5) on the 3’ side will enable transcriptional repression. This can be seen in Figure 4B, where RNAs that contain the pattern SL2-SS3-SL4, SL1-SS3-SL4, or SL2-SS3-SL5 are all functional transcriptional repressors. These data imply that B2 RNA is composed of redundant, modular stem-loops that function in transcriptional repression.

Redundancy in functional domains has been previously observed in other ncRNAs. For example, roX1, an ncRNA involved in dosage compensation in Drosophila, can tolerate deletions across the molecule while still maintaining near normal activity, suggesting redundant functional elements are present [12]. Analogous internal redundancy is also observed within roX2, the other ncRNA that contributes to dosage compensation in Drosophila [13]. roX1 and roX2, together with five proteins, form the complex that mediates dosage compensation by coating the male X chromosome and activating its transcription. *In vivo* studies have revealed that combinatorial mutations in roX stem-loops result in loss of complex formation and dosage compensation, leading to the model that redundant structural domains within ncRNAs might enable flexibility and plasticity in nucleating large ribonucleoprotein complexes [14]. As the list of ncRNAs functional in transcriptional regulation continues to grow, it is likely that others will be found to contain modular and functionally redundant structural regions.

The observation that all B2 RNA constructs that are functional as repressors have an SL‑SS‑SL pattern can be considered in light of our working model for repression. B2 RNA binds Pol II in the cleft normally occupied by DNA, Pol II/B2 RNA assembles into complexes on promoter DNA with general transcription factors, and B2 RNA prevents Pol II from properly engaging the DNA [5,6,7,8]. It is possible that the two stem-loops surrounding the single-stranded region occupy the DNA cleft on Pol II, and the inherent flexibility of the single-stranded region is required for the stem-loops to orient properly in the DNA cleft. Given that the stem-loop in region 1 is substantially larger than that comprising region 2, there must be some flexibility in how B2 RNA is positioned within the cleft.

The observation that repression requires an unstructured region of B2 RNA is consistent with the mechanism by which Alu RNA represses transcription. Alu RNA is a human SINE-encoded ncRNA that also represses transcription by binding directly to Pol II [11]. Alu RNA also contains a loosely-structured region that is important for transcriptional repression. This regions is best described as an extended bubble within a long stem. Considered with the model for transcriptional repression by B2 RNA described here, it seems that repression by either RNA minimally requires an extended flexible region surrounded by two stems. For both B2 RNA and Alu RNA, it remains to be determined whether RNA specific sequences within the critical structures are important for transcriptional repression.

In our past studies of B2 RNA and Alu RNA we found that transcriptional repression and Pol II binding are separable functions. Our current studies support this model; regions in B2 RNA important for transcriptional repression could be separated from regions important for Pol II binding. Indeed, Figure 1, Figure 2 and Figure 3 each show examples of deletion constructs that were impaired in their ability to repress transcription, however, still bound Pol II well. Future work will be needed to delimit the regions of B2 RNA that are critical for binding to Pol II, and to determine if binding is mediated primarily by sequence or RNA structure.

## 4. Material and Methods

### 4.1. Preparation of RNA

The plasmid pUC-T7-B2 was previously described [4]. The plasmids pUC-T7-B2(Δ81-98), pUC-T7-B2(Δ99-113), pUC-T7-B2(Δ116-130), and pUC-T7-B2(Δ154-178) were constructed using the parental plasmid pUC-T7-B2 and performing the QuikChange protocol (QuikChange kit; Life Technologies, Grand Island, NY, USA). Templates for other B2 RNA deletion constructs were made by either PCR or by annealing template and non-template strand oligonucleotides containing the T7 RNA polymerase promoter upstream of the sequence encoding the desired B2 RNA deletion. ^32^P body-labeled or unlabeled B2 RNA constructs were prepared using transcription by T7 RNA polymerase and gel purification, as previously described [4]. Purified RNAs were denatured and folded prior to use by incubating for 3 min at 95 °C in Buffer A (10% glycerol, 10 mM Tris (pH 7.9), 10 mM HEPES (pH 7.9), 50 mM KCl, 4 mM MgCl_2_, 1 mM DTT), and immediately putting them on ice.

### 4.2. *In vitro* Transcription Assays

Recombinant human TBP, TFIIB, TFIIF, and native human Pol II were prepared as previously described [15,16]. Negatively-supercoiled plasmid containing the adenovirus major late promoter (−40 to +10) fused to a 380-bp G-less cassette was used as the DNA template [15]. 20 μL reactions were assembled in Buffer A plus 25 μg/mL BSA. Factors were used at the following final concentrations: 3.5 nM TBP, 10 nM TFIIB, 2 nM TFIIF, 2 nM Pol II, 1 nM DNA template, and 5 nM B2 RNA construct. Reactions were assembled as follows. In 10 μL, TFIIB, TFIIF and Pol II were preincubated at 30 °C for 3 min and folded B2 RNA (full length or deletion construct) was added to the mixture. In a separate tube, DNA template was preincubated with TBP in 10 μL at 30 °C for 3 min. 50 ng of poly-dGdC as a non-specific competitor was added to the tube containing the B2 RNA, TFIIF, TFIIB, and Pol II. The contents of the two tubes were mixed, then incubated at 30 °C for 20 min. 2 μL of a nucleotide triphosphate stock solution was added to the reaction yielding final concentrations of 625 μM ATP, 625 μM UTP, 25 μM (5 μCi alpha-^32^P[CTP]). After 20 min of transcription the reactions were stopped and subjected to denaturing PAGE as previously described [15]. Every experiment that tested B2 RNA deletions also contained control reactions with full-length B2 RNA and no B2 RNA.

The gels were visualized using phosphorimagery and the bands were quantified using ImageJ software. The fold-repression in reactions containing a B2 RNA (full-length or deletion) was calculated by comparison to transcript production in reactions without B2 RNA. The average fold-repression by B2 RNA across all experiments was 7.8. For purposes of plotting, the fold-repression for each sample was divided by −7.8, which set the relative repression by full length B2 RNA to −1.0 and normalized the repression data for the deletions accordingly. The one-sided Dixon's test for single outliers was used to find low and high outliers, which were omitted (only two data points were omitted across all experiments). For statistical comparisons between samples, the fold-repression prior to normalization was used in unpaired *t* tests. The data for each deletion construct were compared to the data for full-length B2 RNA; in separate *t* tests the data for each deletion in the 5’ series and the 3’ series were also compared to the data for its nearest larger neighbor. All two-tailed *p* values < 0.05 were considered to be statistically significant, as indicated by asterisks in the figures.

### 4.3. Electrophoretic Mobility Shift Assays

5 nM ^32^P-labeled B2 RNA or B2 RNA deletion was incubated with 2 nM Pol II for 10 min at 30 °C in 20 μL Buffer A plus 25 μg/mL BSA. 50 ng of poly-dGdC was added and the incubation was continued for 5 min at 30 °C. Complexes were subjected to electrophoresis through 4% native polyacrylamide (37.5:1 acrylamide:bis ratio) gels containing 0.5X TBE, 4% glycerol, and 5 mM magnesium acetate. The gels were visualized using phosphorimagery and the bands quantified using ImageJ software [17]. The amount of RNA bound to Pol II was quantitated and divided by the number of C's in each construct to account for differences in the bound signal due to differential ^32^P-labeling of the RNAs. Within each experiment, the data were then normalized to the amount of bound full-length B2 RNA, setting this value to 1.0 and obtaining fractional binding for each of the B2 RNA deletions. The composite data for full-length B2 RNA from all experiments is shown in each figure. For statistical comparisons between samples, the fractional binding was used in unpaired t tests. The data for each deletion construct were compared to the data for full-length B2 RNA; in separate t tests the data for each deletion in the 5’ series or the 3’ series were also compared to the data for its nearest larger neighbor. All two-tailed *p* values < 0.05 were considered to be statistically significant, as indicated by asterisks in the figures.

## Supplementary Files

Supplementary File 1
