# Supplementary material for: Repression of RNA Polymerase II Transcription by B2 RNA Depends on a Specific Pattern of Structural Regions in the RNA
Source: Noncoding RNA. 2015 Jan 28;1(1):4–16. doi: 10.3390/ncrna1010004 (PMC4578731; doi:10.3390/ncrna1010004)
Supplement: Supplementary File 1 [file ncrna-01-00004-s001.pdf]

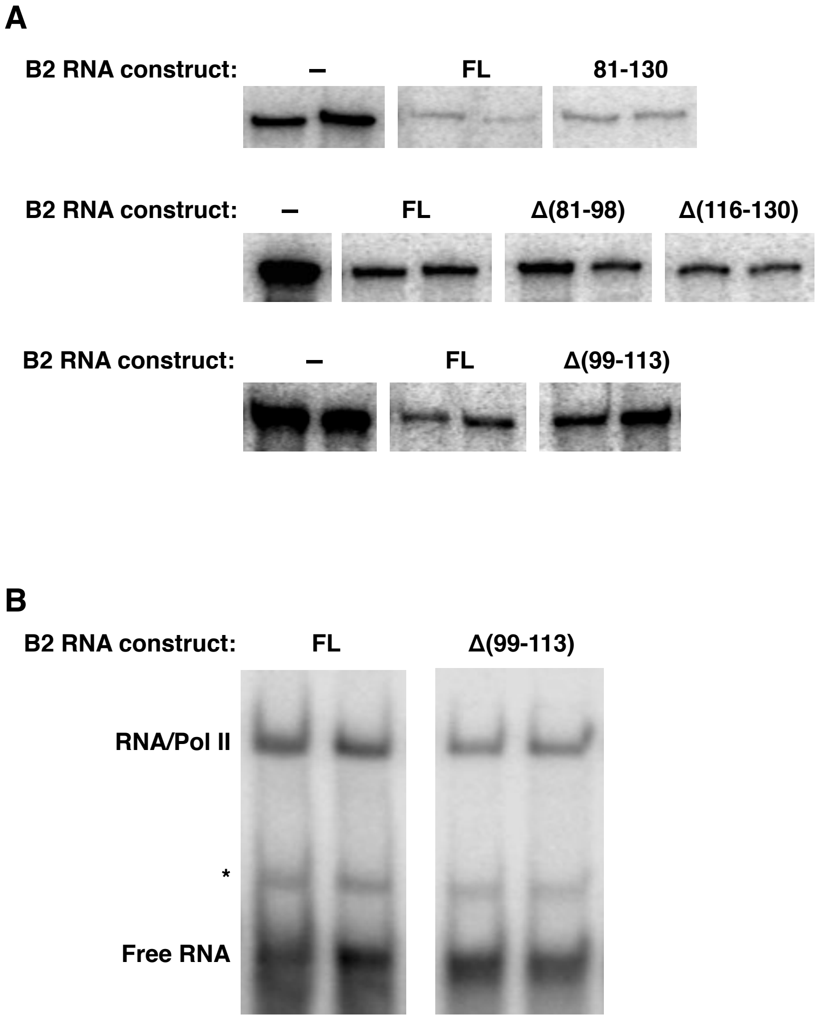

Figure S1. Representative transcription and binding data for the constructs tested in Figure 1B. (A) Transcription data. The ^{32}P bands for G-less RNA transcripts that were resolved on denaturing gels are shown. The data in each row came from a single gel. The B2 RNA construct added to each transcription reaction is indicated above the gel images. Two representative bands are shown for each B2 RNA construct tested in Figure 1B. (B) EMSA data. The gel images show representative EMSA data for the two B2 RNA constructs for which binding assays were performed in Figure 1B. The B2 RNA construct added to each reaction is indicated above the gel images. Two representative lanes are shown for each B2 RNA construct tested in Figure 1B. The positions of the RNA/Pol II complex and the free RNA are indicated. The asterisk indicates the position of a minor alternative conformation of B2 RNA that we sometimes see in our preparations.
